# High Glucose Promotes Pancreatic Cancer Cell Proliferation via the Induction of EGF Expression and Transactivation of EGFR

**DOI:** 10.1371/journal.pone.0027074

**Published:** 2011-11-08

**Authors:** Liang Han, Qingyong Ma, Junhui Li, Han Liu, Wei Li, Guodong Ma, Qinhong Xu, Shuang Zhou, Erxi Wu

**Affiliations:** 1 Department of Hepatobiliary Surgery, First Affiliated Hospital of Xi'an Jiaotong University, Xi'an, China; 2 Department of Pharmaceutical Sciences, North Dakota State University, Fargo, North Dakota, United States of America; The University of Hong Kong, China

## Abstract

Multiple lines of evidence suggest that a large portion of pancreatic cancer patients suffer from either hyperglycemia or diabetes, both of which are characterized by high blood glucose level. However, the underlying biological mechanism of this phenomenon is largely unknown. In the present study, we demonstrated that the proliferative ability of two human pancreatic cancer cell lines, BxPC-3 and Panc-1, was upregulated by high glucose in a concentration-dependent manner. Furthermore, the promoting effect of high glucose levels on EGF transcription and secretion but not its receptors in these PC cell lines was detected by using an EGF-neutralizing antibody and RT-PCR. In addition, the EGFR transactivation is induced by high glucose levels in concentration- and time-dependent manners in PC cells in the presence of the EGF-neutralizing antibody. These results suggest that high glucose promotes pancreatic cancer cell proliferation via the induction of EGF expression and transactivation of EGFR. Our findings may provide new insight on the links between high glucose level and PC in terms of the molecular mechanism and reveal a novel therapeutic strategy for PC patients who simultaneously suffer from either diabetes or hyperglycemia.

## Introduction

Diabetes mellitus and therioma are familiar diseases that tremendously impact human health worldwide. Epidemiologic evidence suggests that patients with diabetes are at a significantly higher risk of developing many types of cancers, particularly cancers of the pancreas, breast, liver, esophagus, and colons [1]. The pancreas is involved in both diabetes mellitus and pancreatic cancer. Diabetes is typically divided into two major subtypes, type 1 and type 2; of these, type 2 diabetes shares many risk factors with cancer. A recent study has demonstrated that approximately 80% of patients with pancreatic cancer (PC) suffer from either hyperglycemia or diabetes, both of which can be detected in the presymptomatic phase of PC [2]. Cancer patients with diabetes are predominantly type 2 in nature [3]. To our knowledge, few studies to date have explored the link between cancer and type 1 diabetes. Furthermore, there is no consensus so far regarding a causal relationship between diabetes mellitus and PC because the nature of the association is believed to be complex. In view of diabetes being associated with an increased risk of PC, it is a fact that large numbers of PC patients suffer from elevated glucose levels. When blood glucose in patients with PC is well-controlled, patient survival time can be prolonged, suggesting that high glucose could directly promote PC progression [4]. Our recent study revealed that high glucose levels promoted cell proliferation through the regulation of expression of glial cell line-derived neurotrophic factor (GDNF) and RET in PC cells [5]. In another study, we demonstrated that hyperglycemia, a common confounding factor associated with PC, may contribute to perineural invasion [6]. However, the mechanism behind this process is still not fully understood.

Epidermal growth factor (EGF) is a low molecular weight (Mr = 6,045) polypeptide that produces hyperproliferation of epidermal tissues when administered to animals [7]. In PC, a variety of growth factors are expressed at elevated levels. Overexpression of EGF and/or TGF-α and EGFR in most PC cells plays a crucial role in PC cell growth [8]. The concomitant presence of EGFR and its ligand, EGF, is associated with enhanced tumor aggressiveness and shorter survival time [9]. The biological functions of cancer cells are remarkably suppressed when specific blockers inhibit EGFR phosphorylation. The EGF-EGFR pathway has recently been discovered as a key therapeutic target in lung cancer. However, there is no study concerning whether glucose concentrations influence the expression of EGF and EGFR in PC.

In addition to its role in binding EGF, EGFR serves a pivotal role as a central transducer of heterologous signaling systems as a result of its transactivation [10]. The transactivation of EGFR by diverse stimuli, such as G protein–coupled receptors, cytokines, or cellular stress, provides a mechanism for the EGFR to integrate these extracellular signals and acts as a relay station to the transcriptional machinery. High glucose has recently been shown to transactivate EGFR in renal disease [11]. However, whether the transactivation of EGFR occurs in PC is not clear.

To investigate how high glucose promotes proliferation of PC cells, we investigated cell proliferation and the expression of both EGF and EGFR in response to increasing glucose concentrations in the PC cells. Through screening, two different differentiation PC cells BxPC-3 (high differentiation) and Panc-1 (low differentiation) were chose in the study. Furthermore, we examined the feasibility of EGFR transactivation in PC,which participates in the proliferation of PC cells.

## Materials and Methods

### Cell culture

Human PC cells BxPC-3 and Panc-1 were purchased from the American Tissue Type Collection ([ATCC], USA). Both cell lines were cultured in Dulbecco's modified Eagle's medium (DMEM) (Life Technologies, USA) supplemented with 10% heat-inactivated fetal bovine serum (FBS) and incubated at 37°C in a humidified atmosphere of 5% CO_2_ in air. Cells were exposed to medium with glucose concentrations varying from 5.5 to 50 mM for 12 h, 24 h, or 48 h to study the effect of glucose concentration.

### Cell proliferation assay

BxPC-3 and Panc-1 cells were seeded in 96-well tissue culture plates at a density of 5,000–10,000 cells per well 24 h prior to serum starvation. After serum starvation for 24 h, cells were cultured in DMEM with concentrations of glucose ranging from 5.5 to 50 mM at 37°C. After 12 h, 24 h, or 48 h, the medium was removed, and MTT reagent (3-(4, 5-dimethylthiazol-2-yl)-2, 5-diphenyltetrazolium bromide) was added to each well. After incubating at 37°C for 4 h, 150 µl of DMSO was added to the cells. Optical densities (OD) at 490 nm were measured using a microplate reader (BIO-TEC Inc, VA). The proliferation rate was defined as OD (cells plate)/OD (blank plate).

### Immunofluorescence

Cells were fixed with 4% paraformaldehyde and then exposed to 3% H_2_O_2_ for 10 min to block endogenous peroxidase. Cells were incubated in nonimmune blocking serum for 15 min to block nonspecific immunoglobulin-binding sites and then incubated with rabbit anti-EGF polyclonal antibody (Sigma-Aldrich Inc.) overnight at 4°C. After washing with PBS 3 times, cells were incubated with fluorescein isothiocyanate (FITC)-conjugated goat anti-Armenian hamster IgG (Jackson Immuno Research) for 1 h at room temperature in a darkroom. After washing with PBS for 5 min and serum-free DMEM for another 5 min, cells were mounted in fluoromount-G (Southern Biotech). As a negative control, the primary antibody was substituted by antibody diluent.

### Transcription-polymerase chain reaction (RT-PCR)

Total RNA from PC cells was extracted using a Fastgen200 Kit RNA isolation system (Fastgen, Shanghai, China) according to the manufacturer's protocol. Total RNA was reverse-transcribed into cDNA using the Fermentas RevertAid™ Kit (MBI Fermentas, Canada). EGF PCR was performed using one cycle of 94°C for 3 min, followed by 35 cycles of 94°C for 30 s, 60°C for 30 s, and 72°C for 35 s. EGFR and β-actin were amplified as follows: after denaturing at 94°C for 3 min, 35 cycles of 94°C for 30 s, 58°C for 30 s, and 72°C for 35 s were employed. Samples were run in triplicate, and the experiment was repeated three times. Gene expression was quantified by normalization to β-actin. The primer sequences were as follows:

β-actin–F: 5′-ATCGTGCGTGACATTAAGGAGAAG-3′.
β-actin–R: 5′-AGGAAGAAGGCTGGAAGAGTG-3′.
EGFR-F: 5′-GGTGGCTGGTTATGTCCTCATTG-3′.
EGFR-R: 5′-AGTTTCTGGCAGTTCTCCTCTCC-3′.
EGF-F: 5′-TGTCTGCGTGGTGGTGCTTG-3′.
EGF-R: 5′-CTGCGACTCCTCACATCTCTGC-3′.

### Western blotting

Proteins were separated on 10% SDS-PAGE gels and transferred to polyvinylidene difluoride membranes. The membranes were blocked with 10% skimmed milk for 2 h at room temperature and then incubated with rabbit anti-human antibodies against EGF or EGFR (Sigma-Aldrich Inc.) or p-EGFR (Cell Signaling) overnight at 4°C. After washing 3 times, the blots were incubated with a goat anti-rabbit peroxidase-conjugated secondary antibody for 1 h at room temperature. Specific binding was detected with the enhanced chemiluminescence system (Sigma-Aldrich Inc.).

### Statistical analysis

Statistical analysis was performed using SPSS software (version16.0, SPSS Inc. Chicago, USA). All data are represented as mean ± standard deviation (SD). Multiple group comparisons were achieved by one-way analysis of variance (ANOVA) followed by the Bonferroni post hoc test. *P*<0.05 was considered statistically significant. All experiments were repeated independently at least three times.

## Results

### High glucose promotes proliferation of PC cells

**Figure 1:**
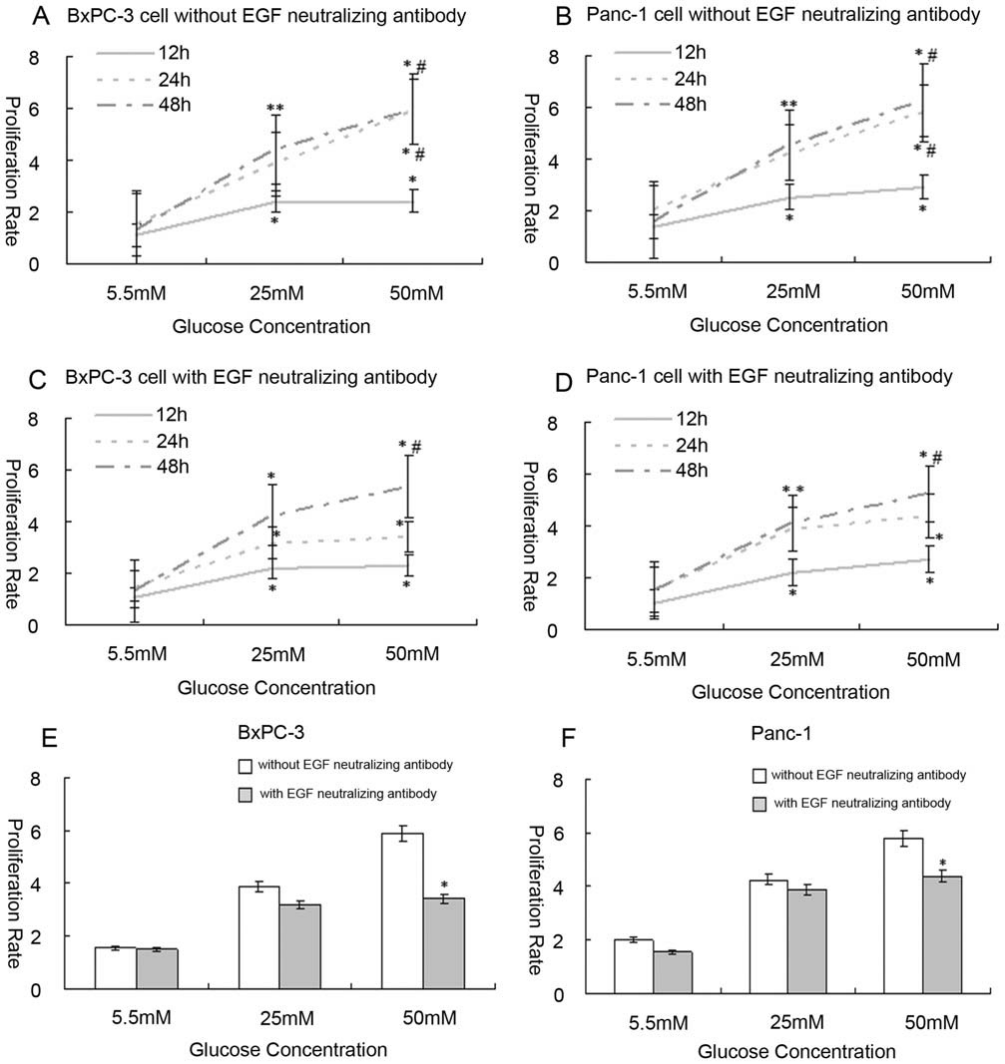
The influence of high glucose on cell proliferation. Cells were exposed to medium with glucose concentrations varying from 5.5 to 50 mM for 12, 24, or 48 h. MTT assay was analyzed for proliferation rates (5.5 mM as a control). (A, B) shows the proliferation rates corresponding to different glucose concentrations in BxPC-3 and Panc-1 cells. (C, D) shows the proliferation rates corresponding to different glucose concentrations in BxPC-3 and Panc-1 cells treated with EGF-neutralizing antibody (* p<0.05 compared with group in 5.5 mM glucose; *^#^ p*<0.05 compared with group in 25 mM glucose; n = 8 per group). (E, F) shows the comparison of proliferation rates before and after treating with EGF-neutralizing antibody with the different glucose concentrations in BxPC-3 and Panc-1 cells at the time point of 24 h (* p<0.05 compared with group of without EGF neutralizing antibody).

To investigate the influence of glucose levels on cell growth, cells were incubated in a series of gradually increasing glucose concentrations for 12 h, 24 h, and 48 h. In BxPC-3 cells and Panc-1 cells, the cell proliferation rate was increased in a dose dependent manner at glucose concentrations of 5.5 mM (as a control), 25 mM, and 50 mM, at 12 h, 24 h, 48 h, respectively (*p*<0.05) (Fig. 1A and Fig. 1B).

We determined the cell proliferation rate after treatment with EGF-neutralizing antibody [12]. As shown in Figure 1C and 1D, increased cell growth of BxPC-3 and Panc-1 cells treated with EGF-neutralizing antibody was observed in response to the glucose concentrations ranging from 5.5 mM to 50 mM (*p*<0.05) (Fig. 1C and Fig. 1D). Comparing to the cells without EGF-neutralizing antibody, cell proliferation decreased by a small margin at glucose concentrations of 5.5 mM and 25 mM, but showed a remarkable drop at 50 mM glucose in the EGF-neutralizing antibody pretreated cells (Fig. 1E and 1F).

### Expression of EGF in pancreatic cancer cells

**Figure 2:**
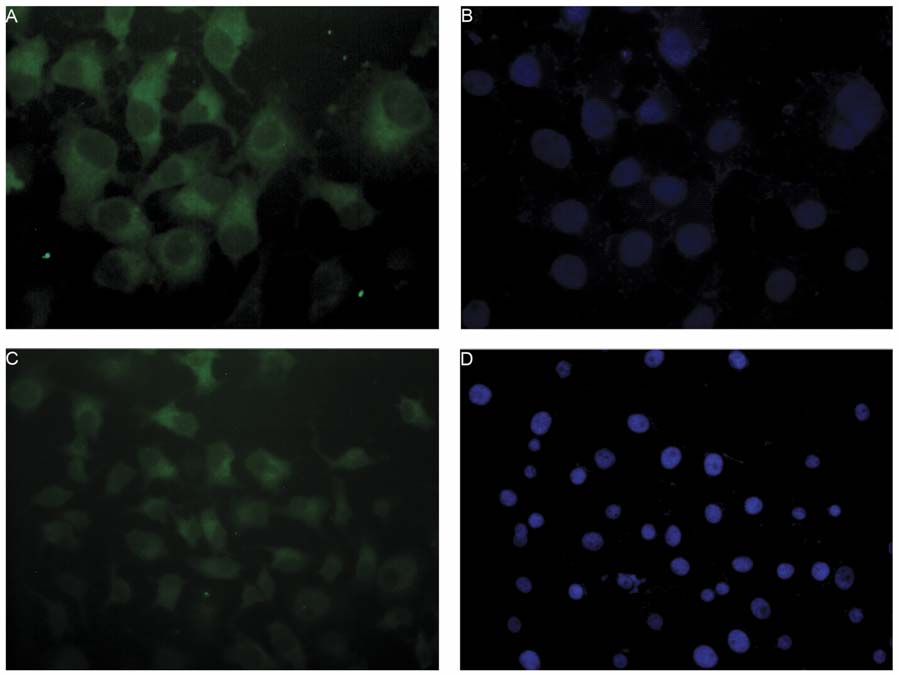
The expression of EGF protein in BxPC-3 and Panc-1 cells. Cells were labeled with fluorescence-conjugated EGF specific antibody (green) in both BxPC-3 (Fig. 2A, 2B) and Panc-1 cells (Fig. 2C, 2D) (200×). Granules with green fluorescence are apparent in cell plasma but not in the nucleus in both BxPC-3 and Panc-1 cells (Fig. 2A and Fig. 2C). Fig. 2B and Fig. 2D were the nucleus staining with DAPI.

It has been demonstrated that EGFR has extensive expression on the cell surface of PC cells [8]. To examine the expression of EGF in BxPC-3 and Panc-1 cells, we used fluorescence-labeled EGF specific antibody to label cells, and the signals were detected using a fluorescent microscopy. Large numbers of immunofluorescence labeled granules were detected in the cytoplasm of both cell lines (Fig. 2). These results demonstrated the expression of EGF in PC cells.

### Induction of EGF mRNA and protein, but no obvious change in the expression of EGFR, under high glucose stimulation

**Figure 3:**
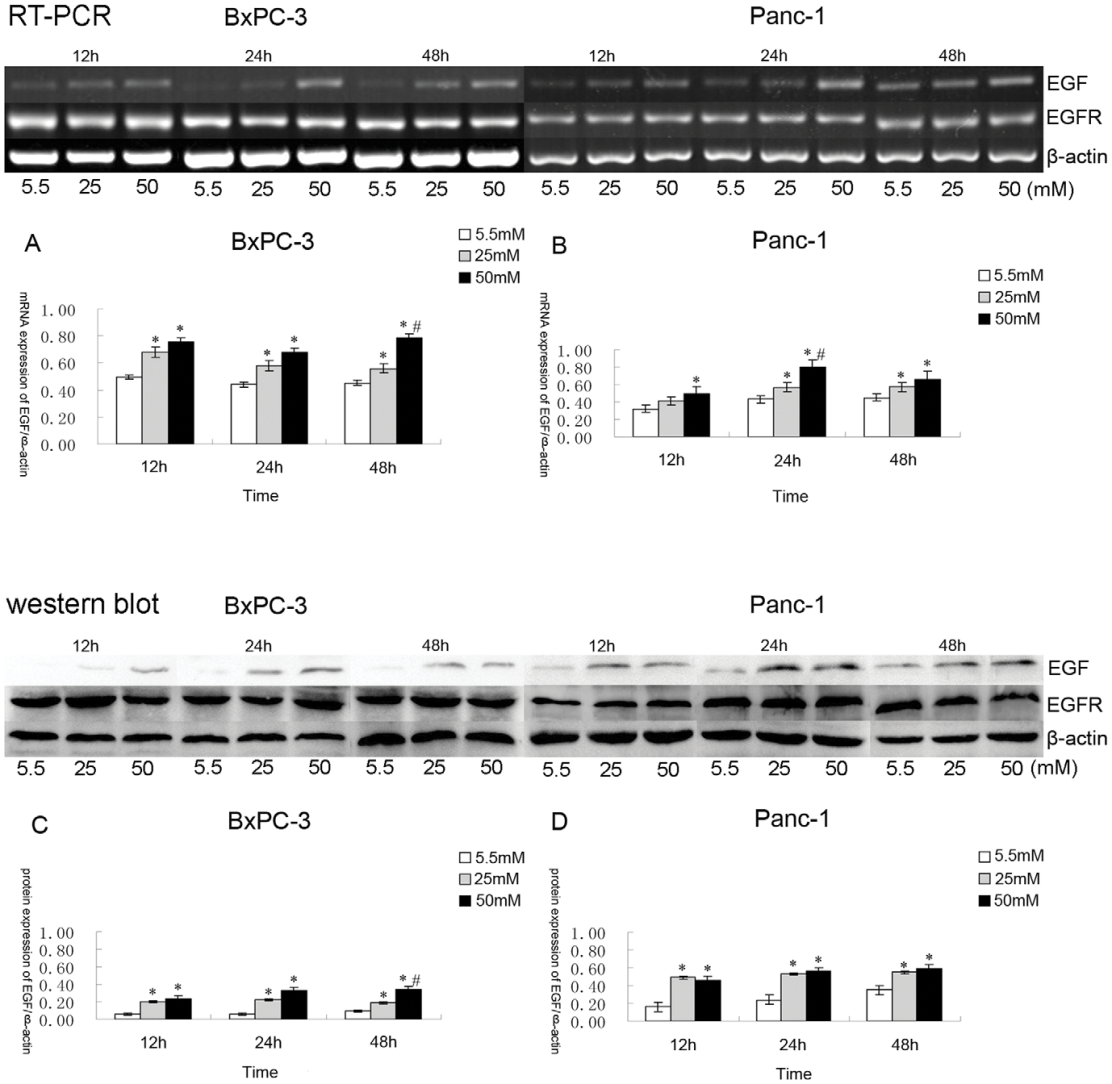
The mRNA and protein expression of EGF and EGFR in BxPC-3 and Panc-1 cells. The expression level of EGF mRNA gradually increased in response to increasing glucose concentrations (5.5 to 50 mM) in culture medium (5.5 mM as a control) (* p<0.05 compared with group in 5.5 mM glucose; *^#^ p*<0.05 compared with group in 25 mM glucose; n = 8 per group). The change was particularly significant between 5.5 and 25 mM glucose. However, the expression of EGFR mRNA maintained a steady remained unchanged (*p*>0.05). (A, B) shows EGF mRNA expression in BxPC-3 and Panc-1 cells; (C, D) shows EGF protein expression in BxPC-3 and Panc-1 cells.

RT-PCR analysis revealed that both EGF and EGFR were expressed in both BxPC-3 and Panc-1 cells (Fig. 3). The expression level of EGF mRNA in both cell lines was up regulated in response to glucose stimulation in a dose dependent manner from 5.5 to 50 mM at all time points (*p*<0.05). The changes between 5.5 and 25 mM were especially significant (Fig. 3A and 3B). However, the expression of EGFR mRNA maintained a steady level irrespective of glucose concentration or time point (*p*>0.05) (data not shown).

The protein expression levels of EGF and EGFR in BxPC-3 and Panc-1 cells were determined by Western blotting (Fig. 3), and the results were consistent with those of the RT-PCR experiments (*p*<0.05) (Fig. 3C and 3D). It is well known that EGF, an essential growth factor, exerts a critical mitogenic effect in both non-malignant and malignant cells [13]. Hence, based on our observation, we reason that the mechanism of action by which high glucose exerts its effects in PC cells might via the upregulation of EGF expression.

### The transactivation of EGFR induced by high glucose

**Figure 4:**
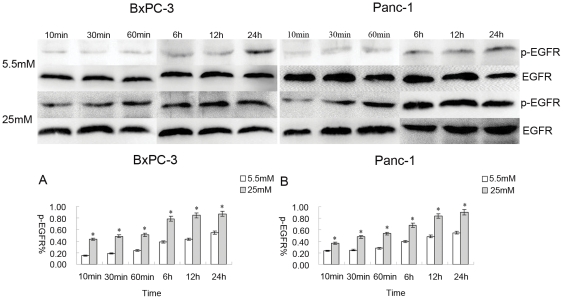
The transactivation of EGFR induced by high glucose. EGF-neutralizing antibody (1 µg/ml) was added to cells before high glucose treatment. The data for p-EGFR % are derived from the ratio of p-EGFR and EGFR. The differences in phosphorylation levels were distinct between 5.5 mM and 25 mM glucose. The phosphorylation level in response to 25 mM glucose increased rapidly in BxPC-3 and Panc-1 cells (Fig. 4). The phosphorylation level in response to 5.5 mM glucose concentration was almost undetectable (10 min, 30 min, and 60 min); it did increase slowly, but it was faint, in BxPC-3 and Panc-1 cells (Fig. 4A and 4B).

The active state of EGFR was assessed by the level of phosphorylation of EGFR. In addition to EGF activating EGFR, other factors, such as HB-EGF, Ang II, and aldosterone, are known to transactivate EGFR [14], [15], [16]. In this study, we determined that high glucose is also among the factors which can induce the transactivation of EGFR. To fully abolish the effect of EGF, cells were treated with 1 µg/ml of EGF-neutralizing antibody before glucose treatment. When BxPC-3 and Panc-1 cells were cultured in the medium containing 25 mM glucose, the phosphorylation level of EGFR gradually increased, in a time dependent manner (10 min, 30 min, 60 min, 6 h, 12 h, 24 h) (*p*<0.05) (Fig. 4). As a control, the phosphorylation levels in the cells were detected in the presence of 5.5 mM glucose. The level of EGFR phosphorylation at 5.5 mM glucose was lower compared to the 25 mM glucose condition in a time dependent manner (10 min, 30 min, and 60 min) (Fig. 4A and 4B).

## Discussion

In this study, we showed that the proliferation of BxPC-3 and Panc-1 cells was affected by different concentrations of glucose in a concentration-dependent manner. We also demonstrated the influence of glucose levels on EGF and its receptors in human PC cell lines. Furthermore, we found that high concentrations of glucose induced a significant increase in EGF transcription and secretion but did not alter the expression of EGFR. In addition, we observed that high glucose induced EGFR transactivation in a concentration- and time-dependent manner in BxPC-3 and Panc-1 cells in the presence of EGF-neutralizing antibody.

PC is among the most deadly of cancers; approximately 75% of patients die within 1 year of diagnosis, and only 5% or less survive for 5 years [17]. Due to this poor prognosis, identification of modifiable risk factors for PC is vital. At present, there is evidence that diabetes and hyperglycemia may be involved in the progression of pancreatic cancer. Although long-standing diabetes is an accepted risk factor for PC, a recent report revealed that diabetes may be caused by PC, and that PC is a diabetogenic state [2]. Currently, the underlying causality between diabetes mellitus and PC has not reached a consensus. In our study, glucose concentration may be an important causal link between diabetes mellitus and PC as hyperglycemia exists in the tumor microenvironment [1], [18], we have studied the influence of high glucose on cell proliferation and investigated the potential mechanism.

Our data indicate that high glucose (25, 50 mM) could significantly increase the proliferation of PC cells compared with low glucose (5.5 mM). The stimulating effect on cell proliferation in PC may be via accelerating cell cycle progression, as occurs in rat vascular smooth muscle cells and human breast cancer cells [19], [20]. That high glucose induced EGF expression suggests there is a pathway for cell proliferation in response to glucose stimulation. However, when cells were pretreated with EGF-neutralizing antibody, the increase in proliferation in response to glucose still occurred (Fig. 1C and 1D). These data indicate that high glucose may promote cell proliferation via pathways apart from EGF. When comparing the proliferation rates of cells with or without EGF-neutralizing antibody treatment, a slight decrease in cell proliferation was found in the presence of an EGF-neutralizing antibody at 5.5 and 25 mM glucose. Significant differences were evident at 50 mM glucose (Fig. 1E and 1F). Therefore, EGF may play a partial role, instead of a sole role, in the effect of low (5.5 mM) or high glucose (25 mM) on cell proliferation. In addition, when cells were cultured in high glucose (50 mM), EGF may play a more important role in the stimulation of cell proliferation than at lower glucose concentrations.

EGF induces hyperproliferation of epidermal tissues and enhances tumor promoting actions such as proliferation and metastasis during the progression of cancer. Therefore, EGF and EGFR have been at the forefront of signaling research and have been targets in the development of therapeutics [9]. EGF and EGFR are widely expressed in malignant cancers, including PC cells. In this study, we determined that the effects of high glucose on cell proliferation occurred via the regulation of EGF and EGFR mRNA and protein levels in PC. We observed that one consequence of high glucose was the increased expression of EGF (Fig. 3), which is highly sensitive to glucose concentration. In contrast to its ligand, EGFR was hardly affected by high glucose. Our results implicated EGF as a potential factor in the effect of high glucose on cell proliferation, but it is unlikely to have played a sole role in this event because other cell factors, such as GDNF and RET [5], and even unknown conditions/factors, could bring positive effects in response to high glucose. The clear relationship between diabetes mellitus and PC, then, still needs further investigation.

Warburg effect, which describes that cancer cells prefer glycolysis over oxidative phosphorylation to generate ATP, is that cancer cells have a higher metabolism by uptake more glucose as compared to normal cells [21]. The alterations on glucose consumption and biosynthetic activity of amino acids, lipids and nucleotides are metabolic changes for sustaining cell proliferation in cancer cells [22]. Oxidative stress plays a vital role in the link between Warburg effect and cancer cells [23]. In addition, some researchers found that uncoupling the Warburg effect from cancer could reduce cancer cell proliferation [21]. So the Warburg effect in cancer cells maybe provide partly understandable network between glucose and PC.

EGFR transactivation via G-protein coupled receptor (GPCR) agonists, such as Ang II, aldosterone [14], [15], β-adrenergic receptors [16], and thrombin [24], has also been particularly well studied. High glucose levels have recently been shown to transactivate EGFR in benign diseases [11]. Transactivation of EGFR by high glucose was observed in PC cells in our study. Glucose concentration may be an important causal link between diabetes mellitus and PC. We used an EGF-neutralizing antibody to inhibit the effect of EGF on EGFR activation. The results showed that the phosphorylation level of EGFR was markedly increased and even occurred earlier when cells were exposed to high concentrations of glucose (Fig. 4). In the present study, we demonstrated the transactivation of EGFR by high glucose in PC cells, but we did not investigate the inner mechanism of transactivation. An early study showed that GPCRs could stimulate metalloproteinases, which induced cleavage of EGF-like ligand precursors, leading to the phosphorylation of EGFR [25]. It has also been suggested that EGFR signaling plays central roles in cancer pathogenesis and progression [26]. Therefore, the growth and survival of cancer cells appear to be sustained by a network of receptors/ligands of the EGF family. We reason that EGFR transactivation may be the main path to induce cell proliferation in response to high glucose. Transactivation of EGFR is an important part of cancer progression, although the process is complex and as yet not fully understood. It is likely that EGFR is a central component in cell signal transduction, and it induces activation of the ras/raf/MEK/MAPK pathway [27], [28], [29] and PI3K [30]. How high glucose affects the relative signaling pathway of EGFR is still not fully understood, and further study is required.

Recently, Ke-Ping Xu et al. reported that high glucose impaired the EGFR–phosphatidylinositol 3-kinase/Akt pathway, resulting in delayed corneal epithelial wound healing [31]. In their studies, high glucose reduced phosphorylation of EGFR likely through reactive oxygen species (ROS). This result seems to run counter to ours. The relationship between high glucose and diseases is rather complicated. High glucose has different actions in dissimilar target organs. For example, high glucose induces cell growth or hyperplasia in pancreatic cancer [5], breast cancer [19] and mesangial cells [11], [32], but reduces cell growth in cornea [31] and prostate cancer [33]. In addition, the mechanism of high glucose on phosphorylation of EGFR is obscure and complicated. For diabetic keratopathy, ROS is a significant and remarkable factor in the course of high glucose impairing the phosphorylation of EGFR, moreover, antioxidants and EGFR ligands shortage are not neglectful factors [31]. Meanwhile, heparin-binding EGF-like growth factor (HB-EGF), which lacks in cornea, may play an important role in the phosphorylation of EGFR by high glucose [31], [32].

### Conclusion

Our results suggest a new avenue for exploring how high levels of glucose may contribute to PC progression. This study may represent a paradigm shift in the relationship between diabetes and PC. Our study found that EGF may play a partial role instead of a sole role, in the influence of high glucose on cell proliferation. Further, we demonstrated that high glucose transactivates EGFR in PC cells. This result informs PC patients of the significance of maintaining stable blood glucose and prompts exploring the underlying mechanisms of the deteriorating effects of high glucose in the two troublesome diseases.
